# Volumetric Alterations of the Cerebral Cortex in Eating Disorders

**DOI:** 10.3390/jcm10235480

**Published:** 2021-11-23

**Authors:** Laura Vidal, Miguel A Ortega, Miguel Angel Alvarez-Mon, Melchor Álvarez-Mon, Guillermo Lahera

**Affiliations:** 1 Department of Medicine and Medical Specialities, University of Alcala, 28801 Alcalá de Henares, Spain; laura.vidal@edu.uah.es (L.V.); maalvarezdemon@icloud.com (M.A.A.-M.); mademons@gmail.com (M.Á.-M.); guillermo.lahera@gmail.com (G.L.); 2 Ramón y Cajal Institute of Sanitary Researcsh, 28034 Madrid, Spain; 3 Cancer Registry and Pathology Department, Hospital Universitario Principe de Asturias, 28806 Alcalá de Henares, Spain; 4 Department of Psychiatry and Mental Health, Hospital Universitario Infanta Leonor, 28031 Madrid, Spain; 5 Immune System Diseases-Rheumatology, Oncology Service an Internal Medicine, University Hospital Príncipe de Asturias, 28806 Alcalá de Henares, Spain; 6 Psychiatry Service, Center for Biomedical Research in the Mental Health Network, University Hospital Príncipe de Asturias, 28806 Alcalá de Henares, Spain

**Keywords:** eating disorders, anorexia nervosa, bulimia nervosa, binge eating disorder, cortical thickness, gray matter, cortical morphometry, voxel-based morphometry, cortical volumetry

## Abstract

Eating disorders are relatively frequent psychiatric disorders that can produce serious consequences at the brain level. In an effort to clarify the neurobiological mechanisms of their pathogenesis, some studies have suggested the existence of modifications of the cortical architecture in eating disorders, but it is unknown whether the alterations described are a cause or consequence of eating disorders. The main objective of this systematic review is to collect the evidence available about the volumetric alterations of the cerebral cortex in eating disorders in adults and their apparent relationship with the pathogenesis of the disease. Initially, 91 articles were found by a search that included the terms anorexia nervosa (AN), bulimia nervosa (BN), binge eating disorder, gray matter, cortical thickness (CT), and brain volume. To pare down the articles, the following inclusion criteria were applied: (1) cortical thickness and/or gray matter volume (GMV) in patients with anorexia, bulimia nervosa, or binge-eating disorder was the main measure of the study; and (2) the sample was adult patients aged 18–65. The exclusion criteria were as follows: (1) articles that did not analyze cortical thickness or gray matter volume; (2) studies with patients with comorbidities; and (3) studies in patients who did not meet the DSM-IV/DSM-V criteria. In the first phase of selection, we proceeded to read the titles and abstracts as a first screen, thereby excluding 62 studies, followed by a complete critical reading of the 29 remaining articles. In this last phase, nine studies were excluded because they did not specify the eating disorder subtype, they included adolescents, or they did not measure GMV or CT. Finally, after the above systematic selection process, 20 articles were included in this review. Despite the methodological heterogeneity of the studies, there was some agreement between them. They showed an overall reduction in GMV in eating disorders, as well as alterations in certain regions of the cerebral cortex. Some of the most often mentioned cortical areas were the frontal, cingulate, and right orbitofrontal cortices, the precuneus, the right insula, and some temporoparietal gyri in cases of AN, with greater cortical involvement in frontotemporal and medial orbitofrontal regions in BN and binge eating disorder. Likewise, certain cortical regions, such as the left inferior frontal gyrus, the precuneus, the right superior motor area, the cingulate cortex, the insula, and the medial orbitofrontal sulcus, often remained altered after recovery from AN, making them potential cortical areas involved in the etiopathogenesis of AN. A reduction in GMV in specific areas of the CNS can inform us about the neurobiological mechanisms that underlie eating disorders as well as give us a better understanding of their possible consequences at the brain level.

## 1. Introduction

Eating disorders, such as anorexia nervosa (AN), bulimia nervosa (BN), and binge eating disorder, are relatively frequent psychiatric disorders among adolescents and young adults, mostly in women. Although their etiology is unknown, obstetric complications and exposure to traumatic experiences in childhood have been investigated as possible risk factors [1,2], as have neurodevelopmental alterations [3].

Eating disorders have systemic effects, including important consequences at the brain level that in turn could be related to the development and progression of the disease. Multiple investigations have focused on these brain alterations by evaluating various parameters, such as gray matter volume (GMV), white matter volume, and total cerebral volume as well as cortical surface area, gyrification, and thickness (CT), among others. In this sense, volumetric anomalies stand out both in the volume of gray matter and in the cortical thickness. Because the studies are performed once the disease is established, it is still unknown whether the alterations described are a cause or consequence of the eating disorder in question.

AN is a neuropsychiatric pathology characterized by a persistent limitation of caloric intake, which leads to a significantly low body mass. It includes an intense fear of weight gain and a distortion of self-perceived body image [4]. In an effort to clarify the different neurobiological mechanisms involved in its pathogenesis, several studies have searched for alterations in brain architecture. These studies have shown alterations in cortical volume and CT, both globally and regionally, with no clear consensus between them.

BN is an eating disorder characterized by recurrent episodes of binge eating and inappropriate compensatory behaviors to avoid weight gain [4]. Binge-eating disorder shares with BN the main symptom of compulsive eating, but unlike BN, it is not followed by compensatory or purgative behaviors. Like any eating disorder, they can cause serious medical complications at the systemic level. Their pathogenesis is also not clear, since the results of the few studies on them are not conclusive.

The main objective of this systematic review is to gather the evidence from the studies published to date to clarify the current knowledge on the volumetric alterations of the cerebral cortex in the different eating disorders in adults.

## 2. Materials and Methods

### 2.1. Search Strategy

The articles reviewed were obtained from the PubMed database from inception until 26 April, 2021. The search strategy used was the following: ((“anorexia nervosa” (MeSH Major Topic)) OR (“bulimia nervosa” (MeSH Major Topic)) OR (“binge eating disorder” (MeSH Major Topic)) OR (“eating disorders” (All Fields)) AND (“gray matter” (MeSH Terms)) OR (“gray matter” (All Fields)) OR (“cortical thickness” (All Fields)) OR (“brain volume” (All Fields))).

The bibliography of each selected study was carefully reviewed, from which two articles were detected that met the selection criteria that were not found in the initial search in the database. After reading these articles, we included both in the review because they met all the inclusion criteria.

### 2.2. Selection Criteria

The selection of articles was based on the following inclusion criteria: (1) CT and/or GMV in patients with AN, BN, or binge-eating disorder was the main measure(s); (2) the sample was adult patients aged 18–65; and (3) the article was written in English. The exclusion criteria used were as follows: (1) articles that did not analyze CT or GMV; (2) studies with patients who presented other metabolic and/or psychiatric pathologies in addition to the eating disorder under study and did not separate the results; (3) studies in patients who did not meet diagnostic criteria for the eating disorder in question according to the DSM-IV/DSM-V; (4) articles that studied the changes in the cerebral cortex, taking into account only BMI without relating it to a specific eating disorder; (5) review articles and meta-analyses; (6) single-case studies; (7) case series; and (8) studies of children, adolescents, or people over 65 years.

### 2.3. Article Selection Process

**Figure 1:**
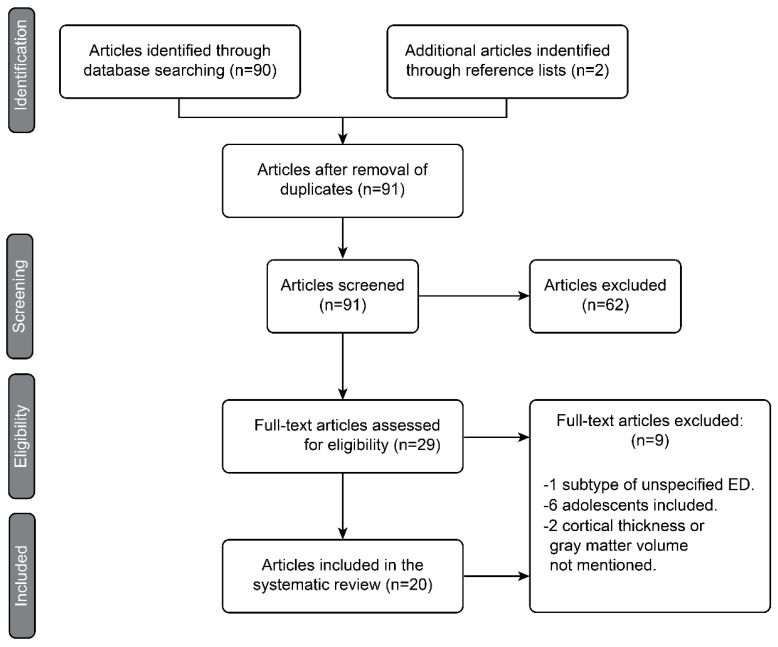
PRISMA diagram representing the article selection process.

Once the articles were identified by the PubMed search and complemented with the manual search, they were pared down in different phases following the guidelines of the Preferred Reporting Items for Systematic Reviews and Meta-Analyses (PRISMA) statement, as shown in the diagram in Figure 1. In the identification phase, duplicate articles were excluded. In the screening phase, all articles that did not meet all the inclusion criteria or met some exclusion criteria were excluded by reading only the titles and abstracts. During the eligibility phase, each full article left after the previous phases was read critically on the basis of the Critical Appraisal Skills Program. The objective of this eligibility phase was to finish excluding the articles that apparently met the inclusion criteria but ultimately did not and to select those studies whose results were valid and applicable for the present systematic review. Finally, in the inclusion phase, 20 articles were selected for the review. We proceeded after this phase to the extraction of data from the studies.

### 2.4. Data Extraction Process for Each Study

To systematize the extraction of information from each study, a table was made with all the data of potential interest, both sociodemographic and related to the results of the study. Sociodemographic data included the number, age, sex, ethnicity or country of origin, BMI, characteristics of the eating disorder, possible associated comorbidities, dominant hand, drug consumption, and educational level of the participants. Regarding the results, we tried to homogeneously collect some variables measured in the studies, both quantitative and qualitative, although not all agreed on the methods or the way the results were expressed. Some examples of these variations are questionnaires of self-perception of the symptomatology, duration of the disease, GMV, CT, qualitative differences of the cerebral cortex between the different groups of participants, and the cortical regions that each study considered of interest for investigating alterations.

## 3. Results

Through the selection process depicted in Figure 1, 20 published studies that met all the inclusion criteria and none of the exclusion criteria were chosen. The main characteristics of the studies are summarized in Table 1 and Table 2.

The selected articles were case–control studies [5,6,7,8,9,10,11,12,13,14,15,16,17,18,19,20,21,22,23] except one study [24], and in all of them, T1-weighted magnetic resonance imaging was used as the imaging technique to assess morphometric and/or volumetric alterations in the volunteers. In most of them, voxel-based morphometry was used [7,9,10,12,14,15,17,18,19,20,21,22,23], an automated technique to evaluate structural changes in the brain that does not depend on the researcher [25]. Other studies used the software FreeSurfer [5,6,8,11,13,24] or machine learning [6].

The studies analyzed included a total of 623 participants with some type of eating disorder. A total of 301 had AN, either restrictive or purgative subtype; 126 of them had BN; 17 patients had binge eating disorder; and 179 participants had recovered from AN (REC). This last group of participants was included in some studies to analyze which of the cortical alterations observed during the acute episode of the disease persisted once normal weight was reached and the baseline situation recovered, as these persistent structural alterations could be trait markers of specific eating disorders. In contrast, it has been postulated that those modifications of the structure of the cerebral cortex that return to normal after the recovery of eating disorder in question could be state markers, that is, of the phase of the disease itself.

### 3.1. Sociodemographic Characteristics of the Participants

In the selected studies, all participants were women, except in one study [18] in which the sample was three men and 23 women. The age distribution by group was homogeneous across all studies. Another variable that was measured in most studies was the laterality or dominance of the hemisphere through the study of the dominant hand of the participants. In some studies [6,9,10,12,21,23,24], all the women were right-handed, while in the rest, either some were left-handed, although always in the minority, or this variable was not measured. The educational level of the participants was also mentioned in some articles [6,7,8,9,10,13,17,23], by number of years studied or by the percentage of participants who had completed secondary school, as well as IQ in two of the studies [7,15]. One of the studies [7] also analyzed the phase of the menstrual cycle which the participants were in at the time of the magnetic resonance imaging. The measurement of all these variables could be necessary to avoid possible biases in future research.

### 3.2. Clinical Characteristics of the Participants

BMI was measured in all the selected studies since it could be important in interpreting whether the results depended on malnutrition in the case of AN or overweight in the case of BN or binge eating disorder. In all studies, a statistically significant difference in BMI was observed between the different groups, the patients with AN having a lower BMI than participants with BN, those with binge eating disorder, healthy controls, or recovered patients.

Likewise, and for similar reasons, the duration of the disease was studied in all women, as was the duration of recovery in the recovered patients. In this aspect, there was a clear lack of homogeneity between the studies, with episodes of disease ranging from 32.86 ± 27.47 months of disease^14^ to 13.2 ± 1 years [13]. There were also differences in recovery times between those studies that included a REC group, from 50.28 ± 19.07 days [16] to 5.2 ± 1.9 years [20].

To find any correlations between the severity of the symptoms and the alterations of the cerebral cortex, most articles gave questionnaires to the participants as a self-assessment of their symptoms. Different versions of different questionnaires were used, such as the Eating Disorder Examination (EDE), EDE Questionnaire (EDE-Q), Eating Disorder Inventory-2 (EDI-2), and EDI-3. Statistically significant variations were observed between patients with eating disorders in the active phase and the REC or the healthy control groups, those with acute disease presenting higher scores for symptomatology on the questionnaires. However, the absence of all types of symptoms in the REC and healthy control groups was required in almost all of the studies.

Information was also collected about the possible comorbidities that the patients could present, such as depression and anxiety, given their high frequency of appearance in this population [5,6,7,9,12,13,14,15,17,18,19,21,24]. Although the vast majority of studies established participants with diagnosed comorbidities as exclusion criteria, questionnaires were used to detect features of depression, such as BDI, BDI-2, HADS, PHQ-9, and QJDS, as well as anxiety traits, such as STAI and HADS. In all the studies that analyzed these comorbidities, to detect possible biases in the results, a statistically significant difference was found in the scores, the anxious-depressive traits being found with greater frequency and greater intensity in the participants with an eating disorder in the acute phase than in the healthy controls and REC. In most studies, it was considered that the results were due to the eating disorder and not to these comorbidities after verifying the similarity of the results when excluding patients with these comorbidities. The same occurred with the patients taking psychotropic drugs such as anxiolytics and antidepressants, although in this case, some studies did find a potential bias [12].

### 3.3. Global Differences in GMV and CT in Eating Disorders

In the present systematic review, two parameters were taken into account to evaluate the integrity of gray matter and its morphological and volumetric alterations in the context of eating disorders: GMV and CT. At least one of these parameters was measured in all the studies included, both at the global level and at the local or regional level.

#### 3.3.1. AN vs. Controls

Several studies found a statistically significant reduction in global GMV in patients with AN compared to the healthy control group [5,7,12,15,16,18,20,21]. In contrast, other studies either did not find any statistically significant difference in GMV between AN and controls [17] or did not study this parameter.

Most studies did not find a significant reduction in CT at the global level, although they did at the regional level in certain regions or clusters [5,15]; if they found such a decrease, they clarified that it was greater at the regional level [18] or partially but not completely global, for example, predominantly in frontotemporal areas [13].

#### 3.3.2. REC vs. Healthy Controls

In those articles that studied the differences in GMV and CT between patients recovered from AN and healthy controls [6,7,8,10,11,13,14,16,20,21,22], different conclusions were found. Many of them found that the differences that were observed in the acute phase of the eating disorder did not persist once the patients recovered from the disease, so in these measures, they achieved complete recovery. These studies suggested that the changes observed in the architecture of the cerebral cortex in the acute phase of the disease could be markers of the state or phase of the disease, since after recovery, they were not observed [7,8,11,13,14].

One of the studies concluded that the brain-morphological recovery was not complete [16].

Other studies found a persistence of cortical alterations in the REC group with respect to controls, but not as much at the global level as at the regional level [6,10,20,21].

One of the studies continued to find cortical alterations in the REC group with respect to the control group, in the form of a persistent reduction in global GMV of approximately 1% in the long term (range of recovery time of the participants from 6 to 60 months) [22].

#### 3.3.3. BN vs. Healthy Controls

No significant differences in GMV were found at the global level in either of the studies that compared BN and healthy participants [9,19].

### 3.4. Regional Differences in GMV and CT in Eating Disorders

**Figure 2:**
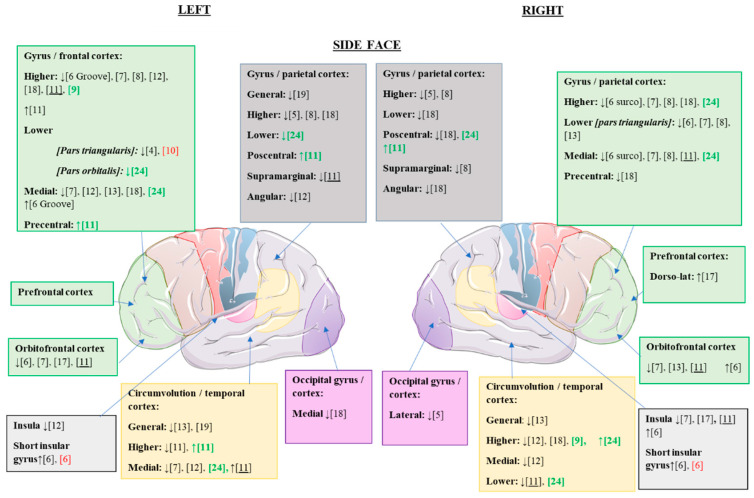
Statistically significant cortical structural alterations in GMV and CT in the different eating disorders compared to control values; lateral view. ↑, increased GMV/CT; ↓, reduced GMV/CT; citations in black, changes observed in patients with AN versus controls; citations in red, changes observed in patients with REC versus controls (possible trait and nonstate changes); citations in green, changes observed in patients with BN versus controls; underlined citations, changes observed in patients with AN versus REC.

**Figure 3:**
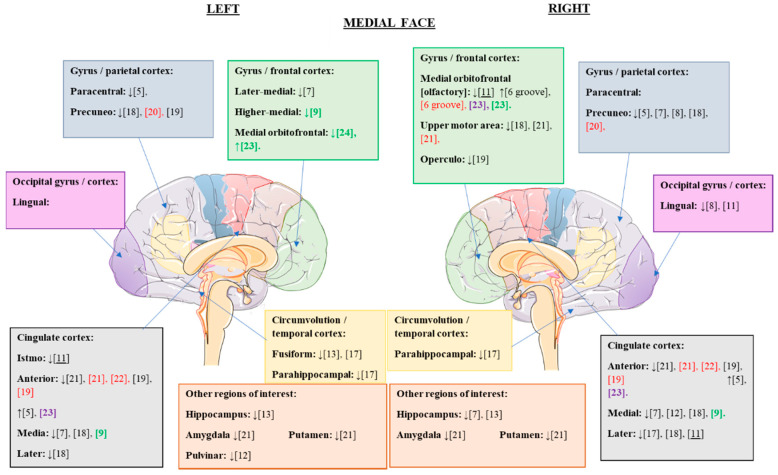
Cortical structural alterations in GMV and CT that were statistically significant in the different eating disorders compared to the control values; medial view. ↑, increased GMV/CT; ↓, reduced GMV/CT; citations in black, changes observed in patients with AN versus controls; citations in red, changes observed in patients with REC versus controls (possible trait and nonstate changes); citations in green, changes observed in patients with BN versus controls; underlined citations, changes observed in patients with AN versus REC; citations in purple, changes observed in patients with binge-eating disorder versus controls.

Once the alterations of the cerebral cortex were analyzed at the global level, we proceeded to study the changes observed in the different articles at the regional cortical level. The cortical alterations found in the different studies are summarized in Figure 2 and Figure 3.

#### 3.4.1. AN vs. Controls

In the present review, statistically significant cortical alterations were found in AN with respect to the healthy control group, as summarized in Table 3 and Table 4.

Kohmura et al. [12] made a distinction between the regions affected after correction for BMI and age and those affected after correction only for age. After correction for BMI and age, a lower GMV was found in participants with AN in the following regions:

- The right superior temporal gyrus, which is involved in body processing, whose alteration can cause some visual dysfunction. A positive correlation was found between the degree of decrease in GMV in this region and the score on the body dissatisfaction questionnaire.
- The right middle temporal gyrus, which is involved in facial recognition, could also cause some visual dysfunction when altered.
- The left pulvinar, involved in facial recognition and selective visual processing, causes distortion of body images when altered.
- The left superior frontal gyrus is involved in inhibitory control.

After correction for age, Kohmura et al. found lower GMV in participants with AN in the following regions:

- Right superior temporal gyrus.
- Left medial temporal gyrus.
- Right medial cingulate, involved in emotions and spatial image processing.
- Left medial frontal gyrus, involved in the spatial image of the body.
- Left angular gyrus.
- Left central operculum.

Brooks et al. [17] made a distinction between the two subtypes of AN. A statistically significant reduction in the GMV was observed in the restrictive subtype compared to the purgative subtype in the bilateral parahippocampal gyrus, in the right anterior insula, and in the left orbitofrontal cortex.

Cascino et al. [11] found that the differences in regional CT between the AN and control groups were not the same as when comparing the AN and REC groups. They obtained the following results:

AN vs. control

- Regions in which a lower CT was found in AN:
  ○ C. superior temporal left
  ○ Right lingual
- Regions in which greater CT was found in AN:
  ○ C. Bilateral superior frontals

AN vs. REC

- Regions in which lower cortical thickness was found in AN:
  ○ Left supramarginal gyrus.
  ○ Bilateral lateral orbitofrontal gyrus.
  ○ Right medial orbitofrontal gyrus.
  ○ Isthmus of the left cingulate gyrus.
  ○ Right posterior cingulate cortex.
  ○ Left superior frontal gyrus.
  ○ Right medial rostral frontal gyrus.
  ○ Right insula.
  ○ Right inferior temporal gyrus.
- Regions in which greater CT was found in AN:
  ○ C. left middle temporal.

#### 3.4.2. AN vs. REC

Only one of the studies compared these two groups, observing a lower GMV in AN than in REC in the left supramarginal gyrus, left superior frontal gyrus, right orbitofrontal medial gyrus, right posterior cingulate gyrus, right inferior temporal gyrus, and right medial rostral frontal gyrus, the bilateral orbitofrontal lateral gyrus, the isthmus of the left cingulate gyrus, and the right insula. Greater GMV was observed in AN than REC in the left middle temporal gyrus [11].

#### 3.4.3. REC vs. Healthy Controls

In the present review, statistically significant cortical alterations were found in AN with respect to healthy controls, as summarized in Table 5.

In studies in which the persistence of these alterations in some cortical regions after recovery from the disease was observed, it was suggested that these alterations could be studied as potential trait markers of eating disorders in future research. That is, by not reestablishing the architecture in these areas, the results suggested that they are independent of the state of the disease [6,10,20,21].

#### 3.4.4. BN vs. Healthy Controls

In the present review, statistically significant cortical alterations were found in BN with respect to healthy controls, as summarized in Table 6.

#### 3.4.5. Binge-Eating Disorder vs. Healthy Controls

Only one study examined cortical alterations in binge-eating disorder, and a statistically significant increase in MVG was observed in the bilateral anterior cingulate cortex and in the right medial orbitofrontal cortex [23].

### 3.5. Correlation of Results with Symptomatology

Multiple studies did not find any correlation between the symptoms of the eating disorder in question, self-assessed by the participants through questionnaires, and the alterations observed with respect to the cortical gray matter [5,7,9,10,15,17,22]. Other studies did observe some association, such as a negative correlation between the GMV of the superior temporal gyrus and the degree of body dissatisfaction [12], between the GMV of the right lower parietal lobe and the drive for thinness in both AN and BN [19], and between the global and regional CT and the score on the DTS-Q questionnaire [24].

### 3.6. Correlation of Results with BMI

While some studies did not find any correlation between gray matter alterations and the BMI of the participants [5,9,15,17,18], others did observe associations. One of these documented a significant direct correlation between GMV and BMI both in the AN and REC groups, suggesting the existence of an inverse relationship between GMV and the degree of malnutrition [7]. Another study did not find a correlation between BMI and GMV when the participants had a low weight, but if a direct relationship was observed between BMI and GMV when the patients began to regain weight, even in the short term, it was not found when GMV had recovered completely [16].

Although some studies did not find a relationship between BMI at the time of the study and GMV, they did find a direct correlation between the lowest BMI throughout life and GMV at the time of the study [14,22]. Specifically, a lower GMV was observed in the precuneus and in both insulae bilaterally in women with the lowest BMI figures throughout their life [14].

### 3.7. Correlation of the Results with the Duration of the Eating Disorder

Not all studies found an association between the duration of the eating disorder and the observed cortical alterations, but some did find a certain relationship [15,16].

Although some did not find an association between these two parameters [5,7,9,12,15,17], a correlation was found between cerebellar atrophy and the duration of the disease that could not be explained by global changes in the GMV [15], in line with previously published studies.

One of the studies reported a negative correlation between the duration of the disease and GMV [16].

### 3.8. Correlation of the Results with Age

In some studies, older participants had a significantly lower cortical volume than the youngest [5,15], while in others, a negative correlation was found between age and the GMV in the medial superior frontal gyrus in women with BN [9].

A negative correlation was also observed between age (which did not differ between groups) and the GMV in the right dorsolateral prefrontal cortex in the healthy controls, which was not observed in the participants with AN [17]. In this study, an increase in GMV was found in women with AN compared to controls in this particular cortical region, suggesting that the central pathology of AN (cognitive restriction of appetitive processes) could avoid the normal effects of atrophy related to age in the right dorsolateral prefrontal cortex and could underlie other cognitive traits of AN, such as inflexible thinking and obsessions about shape, weight, and diet [17].

## 4. Discussion

The main objective of this systematic review was to collect the evidence available to date to clarify the current knowledge regarding the volumetric alterations of the cerebral cortex in the different eating disorders in adults. Through the analysis of the different GMV and CT data reported in the studies reviewed, we found that there was a significant disagreement between them.

### 4.1. Regional Alterations of GMV and CT in Eating Disorders

Despite the differences between the results, some agreement was observed between the studies, mostly in the finding of lower overall GMV in eating disorders [7,12,15,16,18,20,21], as well as in the finding of alterations in certain regions of the cerebral cortex, discussed below.

Lower GMV in the superior frontal gyrus in patients with AN or BN with respect to controls was documented by several articles [6,7,8,9,11,12,18], as well as in the middle frontal gyrus [7,12,13,18,24], predominantly on the left side. In contrast, more gray matter in the left superior frontal gyrus [11] or in the left middle frontal gyrus [6] was observed in the two other studies selected in this review. Likewise, less gray matter has been observed in the inferior frontal gyrus and the right [6,7,8,13] and left pars triangularis [7,10].

The role of the frontal lobes has long been associated with selective attention, working memory, inhibition, and self-control [26]. The superior frontal gyrus is considered a region of inhibitory control; thus, abnormal eating behaviors such as binge eating and purging, as well as restrictive eating, could be derived from alterations in this region [27]. It has been suggested that the superior frontal gyrus plays a role in introspection [28], and lower activation has been previously reported in the AN during self-image processing [29,30]. The medial frontal gyrus, among other functions, deals with visuospatial images of one’s own body [31]. Their deterioration is a potential mechanism causing an altered body image and the need for body weight control [32]. Thus, it has been suggested that a decrease in gray matter in the medial gyrus, possibly caused by the loss of body weight in eating disorders, accelerates and reinforces this clinical condition [12]. Regarding the right inferior frontal gyrus (right pars triangularis), it has been suggested that it is involved in the inhibitory control of inappropriate behaviors [33,34,35].

Clinical and experimental evidence suggests that patients with AN are affected by impaired metacognitive functioning and alexithymic traits, and these dysfunctions in the regulation of thoughts and emotions seem to have a specific role in the maintenance of AN [36,37,38]. Interestingly, the differences in brain volumes tend to be localized, as discussed above, in regions involved in metacognitive and cognitive functioning, such as the dorsolateral prefrontal cortex [17], the superior frontal gyrus [6,7,8,11,12,18], the inferior frontal gyrus [6,7,10,11], and the anterior cingulate cortex [5,9,21,22].

Several included studies also found a smaller left orbitofrontal cortex in AN than the control group [6,7,11,17], while one of the articles documented a greater right orbitofrontal cortex volume [6]. Specifically, a reduction in GMV has been documented in the left orbitofrontal cortex in restrictive AN compared to purgative AN. This is an area associated with specific sensory satiety, and the reduction in its volume can alter the ability of people with AN to feel hunger and appreciate the “appetite” for food [17]. Another study found a greater volume of the left orbitofrontal cortex in people with BN or AT [23].

Alterations in the cingulate cortex were also observed in quite a few studies in AN, BN, and binge-eating disorder. These alterations range from less gray matter in the bilateral anterior cingulate cortices in AN [19,21,22] and bilateral media in AN [7,12,18] and BN [9] to more gray matter in the bilateral anterior cingulate cortex in patients with AN [5] and binge-eating disorder [23] with respect to healthy controls. The cingulate cortex is critical for the generation of emotions and for affective processing [39]. Projecting to the parietal lobe, it is involved in spatial recognition, so its dysfunction, possibly caused by a loss of body weight, can lead to an alteration of spatial perception, leading to a distorted spatial recognition of the body shape [12]. Regarding the increase in CT in the anterior cingulate cortex found in some studies [5,23], this could be related to the possible attempts at compensation or the overexertion made to overcome the difficulties in the regulation of emotions [40,41].

We also observed a substantial preference for involvement of the frontotemporal regions in BN and binge-eating disorder, with less gray matter in the upper, middle, and medial frontal areas [9,24] and more gray matter in the precentral, postcentral, bilateral superior temporal, and medial orbitofrontal gyri [11,23].

### 4.2. Persistence of the Cortical Alterations Observed after the Recovery of Eating Disorders

Only some studies reported persistent alterations in cortical volume and/or thickness after eating disorder recovery [6,10,20,21]. In these articles, the authors postulated that certain regions with persistent cortical alterations observed in the acute phase of an eating disorder could be considered potential trait markers of eating disorders for future research. Similarly, nonpersistent regional alterations after eating disorder recovery could be caused by weight loss itself, creating dysfunction in these cortical regions that collaborates in the perpetuation of the disease [12].

We observed the persistence of lower GMV in the inferior frontal gyrus and left pars triangularis [10], the bilateral precuneus [20], the right superior motor area [21], and the bilateral anterior cingulate cortex [19,21,22], while the persistence of greater CT was documented bilaterally in the short insular gyri and in the medial orbitofrontal sulcus [6]. The right inferior frontal gyrus (right pars triangularis) is involved in the inhibitory control of inappropriate behaviors [33,34,35]. The precuneus might play a central role in visuospatial processing, attention, episodic memory, self-awareness, and mental representations related to oneself [42]. A reduction in insular volume could alter somatosensory processing, which was more pronounced in restrictive AN than in purgative AN [17]. On the other hand, in combination with the reduction of the orbitofrontal cortex, a reduced volume in the insular cortex can alter the ability of people with AN to feel or appreciate hunger [17].

Regarding the causal relationship between the persistent cortical alterations found after recovery and the development of the different eating disorders, Wallace et al. [43] examined 456 adult participants with subclinical features of AN and 247 with subclinical features of BN. To evaluate the subclinical traits, they used the EDI-3 questionnaires for the drive for thinness and for bulimia nervosa (EDIThn and EDIBul, respectively). T1-weighted nuclear magnetic resonance was used to examine cortical alterations. They found a negative correlation between the strength of the drive for thinness self-assessed through the EDI-3 and the CT in the insulae and orbitofrontal cortices, as well as a negative correlation between the degree of presentation of subclinical traits of bulimia according to EDI-3 and the CT of the insulae, bilateral orbitofrontal and inferior parietal cortices, and the left somatosensory cortex. Using these regions as seeds, they also found that their CT and the CT of the left prefrontal and right temporoparietal regions were positively correlated with EDIThn and that the bilateral cingulate and sensory-motor cortex CT was positively correlated with EDIBul. These results coincide with some of those found in the studies included in the present review, such as the persistent reduction in the cerebral cortex in the cingulate [19,21,22] and motor cortices [21] after recovery from AN, although Wallace et al. [43] found these alterations in a preclinical period of BN. In contrast, the latter study suggests a reduction in the cerebral cortex in insular and orbitofrontal areas in subclinical stages of eating disorders, while Lavagnino et al. [6], included in this review, found a persistent increase in cortical thickness in these regions after recovery from AN.

Although we cannot obtain conclusive results to establish the causal relationship between the alteration of certain cortical regions and the development of eating disorders, the finding that areas such as the insular, orbitofrontal, cingulate, and motor cortices were altered in both preclinical and postclinical stages, after recovery, may suggest that these alterations are indeed present before the development of eating disorders in question. Thus, the alterations described in these areas are more likely to be potential risk factors for the development of eating disorders than to be alterations dependent on the state of malnutrition.

### 4.3. Correlation of Cortical Alterations Observed in Eating Disorders with Age

In the present review, only studies in adults were selected because the morphological and volumetric changes that occur in the cerebral cortex in the context of eating disorders follow significantly different patterns between adolescents and adults.

It has been proposed that the brains of adolescents seem to react differently to starvation because the brain is still developing. Gray matter is decreased in acute AN, even more so in adolescents than in adults. In addition, gray matter seems to recover completely in adults, but theoretically, small deficits could persist that have not been detected or that have not reached statistical significance, making the long-term recovery of adolescents completely uncertain [44].

Among the studies selected for the present review, which had only adult participants, the older participants had a significantly lower cortical volume than the younger participants, both overall [5,15] and in more specific regions, such as the medial frontal gyrus. In older women with BN [9] and older women except in the AN group [17], the right dorsolateral prefrontal cortex was normal or larger.

The present systematic review had some limitations in regard to comparing the different findings found in the selected studies. Most of the difficulties were due to the various heterogeneity of the studies. Although heterogeneity in age could have been a problem, we avoided this potential bias by including only studies in adults and leaving studies sufficiently homogeneous in age.

One of the main problems posed by the vast majority of studies was their small samples; future research in this field should propose larger samples to obtain more reliable results than what has been found in the cerebral cortex. Another important limitation was the cross-sectional nature of most studies, so longitudinal studies should be done to track the progression of patients with respect to themselves and to better assess the relationship between the state of the disease and the alterations in the cortex. In addition, longitudinal studies with long-term follow-up could clarify the meaning of the causal relationship, which has been controversial. It is necessary to carry out more studies of this type to determine which cortical alterations are trait markers of eating disorders and therefore predispose patients to suffering from the disease and which alterations constitute state markers and are a consequence of the malnutrition in the acute phase of the disease. Likewise, the differences between the duration of the disease between the selected studies have been an important limitation when assessing the observed results.

Regarding the homogeneity of the sample, the present review includes the variables most commonly measured in the selected studies. Not all collected these data in a homogeneous way, so it would be good for future research to collect them following the same guidelines. It would be interesting if all the studies collected the laterality or dominant hemisphere of the participants or included only people with the same laterality and that the same questionnaires were carried out for the evaluation of the severity of the symptoms and thus told us more precisely and homogeneously the phase in which the disease is found.

It is very important that studies continue to minimize the presence of comorbidities such as depression and anxiety to avoid possible biases, although it could be difficult to obtain a large enough sample if the exclusion criteria with respect to this were stricter. It is also important to exclude, as far as possible, participants taking psychotropic drugs.

## 5. Conclusions

In the present systematic review, the findings documented in the studies found to date regarding the volumetric alterations of cortical gray matter in eating disorders in adults, expressed as reductions or increases in GMV and/or CT, are collected.

Discrepancies were found between the different studies in the alterations that the cerebral cortex presents during the acute phase of eating disorders, as well as after recovery from the disease. However, there is some agreement between the studies, mostly in the reduction in the overall GMV in eating disorders, as well as in the finding of alterations in certain cortical regions more frequently than others. In AN, some of the altered cortical regions about which several studies agreed were the frontal cortex, the cingulate cortex, the precuneus, the right orbitofrontal cortex and insula, and some temporoparietal gyri, among others. Greater cortical involvement could be observed in frontotemporal and medial orbitofrontal regions in the BN and binge-eating disorder. After the recovery from AN, some studies have observed a persistence of cortical alterations, especially in regions such as the left inferior frontal gyrus, precuneus, right superior motor area, cingulate cortex, insula, and medial orbitofrontal sulcus. In contrast, other studies ensure a complete recovery after weight restitution and disappearance of symptoms, keeping in mind that the cortical alterations that depend on the state of the disease, although they can promote the persistence of the disease over time, seem to be more of a consequence than the cause of eating disorders.

The alterations in areas such as the insular, orbitofrontal, cingulate, and motor cortices in both preclinical and postclinical stages (after recovery) may suggest that these regions are effectively present before the development of eating disorders in question. Thus, the alterations described in these areas are more likely to be a potential risk factor for the development of eating disorders than to be alterations dependent on the state of malnutrition. Future research could focus on the study of these regions to clarify this causal relationship.

The reduction in GMV in specific areas of the brain can inform us about the neurobiological mechanisms that underlie eating disorders, as well as about their possible consequences at the brain level.

To clarify and expand the current knowledge on cortical alterations in eating disorders and their causal relationship, it will be necessary to continue investigating these alterations through longitudinal studies, with larger and homogeneous samples in terms of age, sex, and duration of the disease, excluding as far as possible participants with associated comorbidities or taking psychotropic drugs.

## Figures and Tables

**Table 1:** Sociodemographic and clinical data (I).

Study	Group			Age, Mean ± σ (Range)			BMI			Disease Duration	Recovery Duration
	AN	REC	HC	AN	REC	HC	AN	REC	HC		
Leppanen et al. [5]	46		54	27.51 ± 9.24		26.35 ± 4.47	15.73 ± 1.41		21.49 ± 1.97	11.39 ± 9.22	
Lavagnino et al. [6]	19	24	24	23.1 ± 5.8	30.3 ± 8.1	27.4 ± 6.3	16 ± 1.1	20.8 ± 2.4	21.6 ± 1.3		
Nickel et al. [7]	31R, 3P	24	41	23.8 ± 4.3	27.1 ± 7	23.6 ± 3.8	16.1 ± 1.4	20.6 ± 1.3	22.3 ± 2.4	AN 6.6 ± 3.7 y REC 7.2 ± 4.7 y	4.25 ± 5.7 y
Favaro et al. [8]	32R, 6P	20	38	26.1 ± 7.2	26.3 ± 7.1	25.3 ± 6.3	15.8 ± 1.8	19.6 ± 1.6	21.7 ± 2.9	AN 78.6 ± 81.3 mo REC 45.7 ± 65 mo	45.4 ± 46.8 mo
Oliva et al. [10]		15	15		25.87 ± 6.15	25.2 ± 1.01		20.1 ± 2.04	21.32 ± 2.45	REC 38.4 ± 38 mo	74.7 ± 67 mo
Kohmura et al. [12]	7R, 13P, 3NS		29	28.5 ± 6.7		28.2 ± 7	13.2 ± 1.5		21.5 ± 3.3	10.5 ± 6.2 y	
Miles et al. [13]	8R, 15P	17R, 7P	24	29.6 ± 8.2	27 ± 6.1	25 ± 6.7	16 ± 1.4	20.9 ± 1.8	22.3 ± 2.2	AN 13.2 ± 1 y REC 9.6 ± 1 y	
Bang et al. [14]		22	22		27.32 ± 5.14	26.14 ± 4.64		20.39 ± 1.66	21.85 ± 1.76	32.86 ± 27.47 mo	51.62 ± 42.70 mo
Fonville et al. [15]	22R, 6P, 3NE		31	23 ± 10		25 ± 4	15.8 ± 1.4		21.8 ± 1.8	7 ± 10	
Roberto et al. [16]	14R, 18P	14R, 18P (Longit.)	21	26.91 ± 6.41		25 ± 3.18	16.03 ± 1.59	20.01 ± 0.59	20.82 ± 1.22	10.15 ± 6.23 y	50.28 ± 19.07 d
Brooks et al. [17]	8R, 6P		21	26 ± 1.9		26 ± 2.1	15.6 ± 0.4		21.4 ± 0.5	9.2 ± 1.9 y	
Bär et al. [18]	26		26	22.96 ± 4.97		24 ± 1.92	16.97 ± 1.46		21.72 ± 1.5		
Joos et al. [20]	12	5	18	25 ± 4.8	29.6 ± 5.1	26.9 ± 5.7	16 ± 1.2	19.9 ± 1.5	21.2 ± 2	7.2 ± 6 y	5.2 ± 1.9 y
Friederich et al. [21]	12	13	14	24.3 ± 6.2	25 ± 4.8	25.6 ± 3.7	15.9 ± 1.6	19.5 ± 1.4	21.1 ± 1.5	AN 6.3 ± 4.4 y REC 5.7 ± 3.6 y	
Mühlau et al. [22]		22	37		(18.4–40.8)	(18.3–40.2)		(17–22.8)	(18.3–24.8)	(1–23) y	(6–60) mo
Cascino et al. [11]	18R, 4P BN 24	10	35	AN 28.63 ± 9.76 BN 27.2 ± 7.07	25.5 ± 6.65	26.77 ± 5.24	AN 16.37 ± 1.56 BN 22.33 ± 2.88	19.82 ± 1.47	21.13 ± 1.96	AN 12.46 ± 8.63 y REC 5.33 ± 4.67 y BN 8.31 ± 7.48 y	
Joos et al. [19]	AN 12 BN 17		18	AN 25 ± 4.8 BN24.5 ± 4.8		26.9 ± 5.7	AN 16 ± 1.2 BN 21.1 ± 2.5		21.2 ± 2	AN: 4.7 ± 3.6 y BN: 7.5 ± 5.7 y	
Li et al. [9]	34		34	22.85 ± 3.89		22.26 ± 2.53	20.46 ± 2.80		20.52 ± 1.52	2.92 ± 2.35 y	
Westwater et al. [24]	37			22.6 ± 4.13 (18–38)			23.9 ± 3.1 (19.4–31.2)				
Schafer et al. [23]	BN 14 BED 17		19	BN23.1 ± 3.8 TA26.4 ± 6.4		22.3 ± 2.6	BN 22.1 ± 2.5 BED 32.2 ± 4		21.7 ± 1.4	BN: 7.3 ± 3.6 y BED: 6.8 ± 4 y	

AN: anorexia nervosa; BN: bulimia nervosa; BED: binge-eating disorder; NS: nonspecific; REC: recovered from AN; HC: healthy controls; R: restrictive type; P: purgative type; Longit.: longitudinal.

**Table 2:** Clinical data (II).

Study	Depression Z Score			Anxiety Z Score			Symptoms		
	AN	REC	HC	AN	REC	HC	AN	REC	HC
Leppanen et al. [5]	0.80 ± 0.83		−0.77 ± 0.30	0.84 ± 0.67		−0.82 ± 0.39	EDE-Q 4.01 ± 1.01		EDE-Q 0.54 ± 0.51
Lavagnino et al. [6]	2 ± 10.5	3 ± 12.5		4 ± 21.1	4 ± 6.7				
Nickel et al. [7]	BDI-II 20.5 ± 10.4	BDI-II 6 ± 5.9	BDI-II 2.5 ± 3.4	STAI 38.6 ± 6.6	STAI 35.3 ± 5.3	STAI 32.6 ± 4.7	EDE 3.2 ± 1.1 EDI-2 61.2 ± 9.4	EDE 0.6 ± 0.4 EDI-2 46.9 ± 4.8	EDE 0.4 ± 0.3 EDI-2 44.5 ± 3.5
Favaro et al. [8]							EDI-3 higher DT & and BD		
Oliva et al. [10]									
Kohmura et al. [12]	BDI 25.3 ± 7		BDI 4.3 ± 4.5				EDI-II thin 9.6 ± 6.9 EDI-II body 12.8 ± 4.2		EDI-II thin 3.2 ± 4.3 EDI-II body 8.8 ± 7.1
Miles et al. [13]	DTL 19 yes 4 no	DTL 9 yes 15 no		ALV 15 yes 8 no	ALV 15 yes 9 no		EDE-Q 4 ± 1.3	EDE-Q 2.3 ± 1.7	
Bang et al. [14]		BDI 6.36 ± 7.94	BDI 1.77 ± 2.69		STAI state 32.14 ± 8.16	STAI state 25.86 ± 5.21		EDE-Q 0.84 ± 0.74	EDE-Q 0.19 ± 0.17
Fonville et al. [15]	HADS dep 11 ± 7		HADS dep 0 ± 2	HADS anx 15.3 ± 3.4 OCI-R 25.6 ± 13		HADS anx 4.6 ± 3.1 OCI-R 4.5 ± 3.3	EDE-Q 4 ± 1.3		EDE-Q 0.6 ± 0.6
Roberto et al. [16]	-	-	-	-	-	-	-	-	-
Brooks et al. [17]	HADS dep 10.3 ± 1.2		HADS dep 1.6 ± 0.4	HADS anx 13.9 ± 0.8		HADS anx 4.4 ± 0.6	EDE-Q restriction 3.0 ± 0.5 Vomiting/mo 5.8 ± 1.6		EDE-Q restriction 1 ± 0.2
Bär et al. [18]	BDI 20.00 ± 12.05		BDI < 5				EDI-2 32.7 ± 11.4		
Joos et al. [20]							EDI-2 higher DT & BD		
Friederich et al. [21]	PHQ-9 10.8 ± 7.4	PHQ-9 4.9 ± 5	PHQ-9 2.7 ± 1.5	HADS anx 9.1 ± 3.5	HADS anx 6.5 ± 4.2	HADS anx 3.1 ± 2.2	EDI-2 313.1 ± 82.5	EDI-2 249.5 ± 72.1	EDI-2 177.3 ± 38
Mühlau et al. [22]									
Cascino et al. [11]							EDI higher DT & BD		
Joos et al. [19]	BDI AN:26.5 ± 11.8 BN: 8.2 ± 10.1		BDI 5 ± 4.6				EDI-2 higher DT & BD		
Li et al. [9]	9 mild symptoms			9 mild symptoms			HCES 33.24 ± 3.56		
Westwater et al. [24]	QIDS 8.16 ± 5.56			TAI 46.3 ± 11.35			EDE-Q total 2.28 ± 1.53		
Schafer et al. [23]							EDI higher DT & BD		

AN, anorexia nervosa; BN, bulimia nervosa; BED, binge-eating disorder; REC, recovered from AN; HC, healthy controls; STAI, State-Anxiety Inventory; BDI, Beck Depression Inventory; DTL, depression throughout life; ALV, anxiety throughout life; HADS, Hospital Anxiety and Depression Scale; dep, depression; anx, anxiety; OCI-R, Obsessive Compulsive Inventory–Revised; PHQ, Patient Health Questionnaire; QIDS, Quick Inventory of Depressive Symptomatology; TAI, Test Anxiety Inventory; EDE-Q, Eating Disorder Examination Questionnaire; EDI, Eating Disorder Inventory; DT, drive for thinness; BD, body dissatisfaction; Longit., longitudinal. Also measured BD.

**Table 3:** Volume of gray matter in anorexia vs. healthy controls.

Study	GMV AN (mL)	GMV HC (mL)
Nickel et al. [7]	678.6 ± 60.4	721.7 ± 55
Kohmura et al. [12]	572.4 ± 41.3 (42% vol total)	634.2 ± 44.9 (46.6% vol total)
Fonville et al. [15]	804.637 ± 470.37	833.791 ± 381.15
Roberto et al. [16]	647.63 ± 62.07	679.93 ± 53.31
Bär et al. [18]	646.66 ± 57.59	701.07 ± 61.63
Friederich et al. [21]	692.8 ± 59.6	752 ± 71.9
Joos et al. [20]	(GM tissue/total intracranial tissue)	(GM tissue/total intracranial tissue)
	0.478 ± 0.018	0.498 ± 0.017

GMV, gray matter volume; AN, anorexia nervosa; HC, healthy controls; GM, gray matter.

**Table 4:** Statistically significant cortical alterations in anorexia vs. healthy controls.

Study	GMV/CT	Regions with AN < HC	Regions with AN > HC
Leppanen et al. [5]	CT	C. Bilateral superior parietal.	C. anterior cingulate.
		Left paracentral parietal.	
		Right precuneus.	
		C. right occipito-lateral.	
Lavagnino et al. [6]	CT	C. Left lateral orbital.	C. Right orbital.
		S. right medial frontal.	S. left medial frontal.
		C. right inferior frontal.	S. Right medial orbital (olfactory).
		S. Bilateral superior frontal.	C. Bilateral short insular.
Nickel et al. [7]	GMV	C. left medial frontal.	
		C. right frontal inferior.	
		C. Orbitofrontal.	
		C. Superior frontal.	
		C. temporal and parietal.	
	CT	C. Bilateral superior frontal.	
		C. Bilateral medial frontal.	
		C. frontal posterior medial left.	
		C. inferior frontal left (*pars triangularis*).	
		C. temporal medial left.	
		C. superior parietal left.	
		Bilateral medial cingulate.	
		Right precuneus.	
		Right insula.	
Favaro et al. [8]	CT	C. Bilateral superior parietal.	
		C. Bilateral superior frontal.	
		Right precuneus.	
		C. right frontal inferior.	
		C. right medial frontal.	
		C. lingual right.	
		C. supramarginal right.	
Kohmura et al. [12]	GMV	C. temporal superior right.	
		C. right temporal medial right.	
		C. left temporal medial.	
		Left pulvinar left.	
		C. right front superior.	
		Right medial cingulate.	
		C. left angular.	
		Left central operculum.	
Miles et al. [13]	CT	C. left medial-rostral frontal.	
		C. right frontal inferior.	
		C. right lateral orbitofrontal.	
		C. left fusiforme.	
Fonville et al. [15]	CT	*Results included in Leppanen* et al. [5]	
Brooks et al. [17]	GMV	C. Bilateral parahippocampal (0.77 mL diff)	Right dorsolateral prefrontal C. (0.59 mL diff).
		Right anterior insula (0.52 mL diff)	
		C. fusiforme left (0.52 mL diff)	
		Right posterior cingulate (0.42 mL diff)	
Bär et al. [18]	GMV	Bilateral posterior medial cingulate.	
		C. right ventral posterior cingulate.	
		Right anterior precuneus.	
		Supplementary motor area.	
	CT	C. Bilateral superior frontal.	
		C. left frontal medial.	
		C. left occipital medial.	
		Lower right parietal lobe.	
		Left superior parietal lobe.	
		C. right angular.	
		C. right postcentral.	
		C. right precentral.	
		Left posterior cingulate.	
		Left precuneus.	
		C. right upper temporal.	
Friederich et al. [21]	GMV, CT	Left anterior cingulate.	
		AN: 6.1 ± 0.86 mL	
		HC: 7 ± 0.75 mL	
		C. anterior cingulate right.	
		AN: 5.5 ± 0.84 mL	
		HC: 6.4 ± 0.84 mL	
		Supplementary motor area right.	
		AN: 6.1 ± 0.68	
		HC: 7.3 ± 0.79 mL	
Joos et al. [19]	GMV	C. Bilateral anterior cingulate.	
		Right frontal operculum.	
		Left parietal cortex.	
		Left temporal cortex.	
		*Left precuneus (not statistically significant but p < 0.1)*	
Cascino et al. [11]	CT	C. left superior temporal.	C. left superior frontal.
		AN: 3.10 ± 0.29 mm	AN: 2.74 ± 0.24 mm
		HC: 3.34 ± 0.23 mm	HC: 2.72 ± 0.22 mm
		C. right lingual.	C. right superior frontal.
		AN: 1.88 ± 0.21 mm	AN: 2.71 ± 0.25 mm
		HC: 1.96 ± 0.19 mm	HC: 2.59 ± 0.26 mm

C., gyrus or cortex; S., sulcus; GMV, gray matter volume; CT, cortical thickness; AN, anorexia nervosa; HC, healthy controls.

**Table 5:** Statistically significant cortical alterations in recovered anorexia patients vs. healthy controls.

Study	GMV/CT	Regions with REC < HC	Regions with REC > HC
Lavagnino et al. [9]	CT		S. Right medial orbital (olfactory).
			C. Bilateral short insula.
Oliva et al. [10]	GMV	C. left inferior frontal.	
Joos et al. [20]	GMV	Precuneus, long-term (>5 year).	
Friederich et al. [21]	GMV, CT	Left anterior cingulate.	
		REC: 6.2 ± 1 mL	
		HC: 7 ± 0.75 mL	
		C. right anterior cingulate.	
		REC: 5.6 ± 0.84 mL	
		HC: 6.4 ± 0.84 mL	
		Right supplementary motor area.	
		REC: 6.6 ± 0.72	
		HC: 7.3 ± 0.79 mL	

C., gyrus or cortex; S., sulcus; GMV, gray matter volume; CT, cortical thickness; REC, recovered group; HC, healthy controls.

**Table 6:** Statistically significant cortical alterations in bulimia nervosa vs. healthy controls.

Study	GMV/CT	Regions with BN < HC	Regions with BN > HC
Cascino et al. [11]	CT		C. Bilateral superior temporal bone.
			C. left paracentral.
			C. left precentral.
			C. left postcentral.
Li et al. [9]	GMV	C. left superior frontal medial.	
		C. right Upper temporal.	
		C. bilateral medial cingulate and paracingulate.	
		C. left superior frontal dorsolateral.	
Westwater et al. [24]	CT	C. left temporal medial.	
		C. left frontal medial caudal.	
		C. right frontal medial rostral.	
		C. right postcentral.	
		C. right inferior temporal.	
		C. right superior frontal.	
		Left medial orbitofrontal.	
		Left Pars orbitalis.	
		C. left inferior parietal.	
Schafer et al. [23]			Bilateral medial orbitofrontal.

C., gyrus or cortex; S., sulcus; GMV, gray matter volume; CG, cortical thickness; BN, bulimia nervosa; HC, healthy controls.

## Data Availability

Not applicable.

## Abbreviations

ED	eating disorder
AN	anorexia nervosa
BN	bulimia nervosa
GMV	gray matter volume
CT	cortical thickness
REC	recovered
BMI	body mass index
